# Exploring the relationship between domain-specific self-efficacy and motivation among university students: a systematic review (2019–2024)

**DOI:** 10.3389/fpsyg.2025.1702507

**Published:** 2025-11-25

**Authors:** Luoting Lin, Mansor Bin Abu Talib

**Affiliations:** 1 Faculty of Social Sciences and Liberal Arts, UCSI University, Kuala Lumpur, Malaysia; 2 Wellbeing Research Centre, UCSI University, Kuala Lumpur, Malaysia

**Keywords:** domain-specific self-efficacy, student motivation, higher education, systematic review, learning outcomes

## Abstract

The interplay between domain-specific self-efficacy and motivation in higher education has garnered significant attention, particularly in the context of challenges such as the COVID-19 pandemic that have profoundly altered the educational landscape. This study provides a systematic review of the literature on the relationship between domain-specific self-efficacy and motivation among university students, adhering to the Preferred Reporting Items for Systematic Reviews and Meta-Analysis (PRISMA) guidelines. From an initial pool of 5,890 articles sourced from the Education Resources Information Centre (ERIC), Scopus, and Web of Science (WoS) databases, 31 studies published between 2019 and 2024 were analyzed. Our findings underscore a predominantly positive correlation between domain-specific self-efficacy and motivation. Higher levels of self-efficacy are consistently linked to increased motivation, suggesting that enhancing self-efficacy could be a crucial strategy for boosting student motivation and, thus, academic success. Notably, many studies focus on Asian regions, highlighting both the universality and cultural specificity of these findings. However, the presence of an outlier study that found no significant relationship between self-efficacy and motivation indicates the complexity of these constructs and the potential impact of situational factors, warranting further investigation. Additionally, this review reveals a growing academic interest in self-efficacy and motivation, particularly within social sciences and education. This trend underscores the importance of a multidisciplinary approach to understanding the intricate dynamics between self-efficacy and motivation. The documented rise in research attention reflects a growing recognition of the critical role that self-efficacy and motivation play in student learning outcomes. In conclusion, this systematic review not only highlights the significant positive impact of domain-specific self-efficacy on student motivation but also calls for more nuanced studies. Future research should explore under-represented contexts and examine the broader implications of these findings across diverse educational and cultural settings.

## 1 Introduction

In the 21st century, characterized by economic globalization, the competition to attract and retain individuals with diverse skills and comprehensive qualities has intensified. This phenomenon essentially represents a competition in educational quality, highlighting the critical importance of cultivating well-rounded talents. During the COVID-19 pandemic, the shift to remote learning posed significant challenges for students, including social isolation, technological barriers, and a lack of direct teacher-student interaction. These challenges have had a profound impact on students’ self-efficacy and motivation, particularly in online learning environments. Many students faced significant challenges, including social isolation, interruptions in practical training, and a noticeable decline in motivation to engage in academic activities (Rahm et al., 2021). As online learning became more prevalent, students with lower technological self-efficacy struggled more with maintaining motivation, especially in the absence of face-to-face interactions and immediate teacher support. Following the outbreak of COVID-19, a study by Zaccoletti et al. (2020) revealed a significant decline in learning motivation among students in Italy and Portugal. Similarly, Usher et al. (2021) found that over 75% of the 358 student respondents at a southeastern university in the United States experienced decreased learning motivation. In a 2023 study conducted in China, Ma and Cheng reported that approximately 30.86% of participants identified a lack of motivation during independent study sessions as their primary challenge. Despite the diminishing direct impact of the pandemic, the global decline in student learning motivation is not expected to recover swiftly in the near term.

## 2 Literature review

### 2.1 Motivation

Motivation, as a multifaceted construct (Pintrich, 2003), is essential to learning. This intrinsic force drives individuals to engage in activities and make choices, resulting from a deliberate behavioral process. In higher education, motivation is particularly critical as it fosters student initiative and proactivity in a relatively autonomous environment. Gardner et al. (1985), in his “Sociopsychological Model,” defines learning motivation as a psychological drive that compels individuals to invest time and effort in their personal goals, reflecting a genuine eagerness and strong desire to acquire specific knowledge. Brophy (1991) further explains that motivation includes students’ perception of the significance and value of the learning process, as well as their aspiration to achieve specific outcomes through their studies. Ryan and Deci (2000) argue that motivation is driven by both internal forces and external stimuli that ignite and sustain a learner’s eagerness to acquire knowledge or skills. They differentiate between intrinsic motivation, which stems from the inherent enjoyment of learning, and extrinsic motivation, which is propelled by external rewards or consequences. Tschannen-Moran and Hoy (2001) describe learning motivation as the inclination to seek understanding in learning activities and to strive to benefit from these experiences. These perspectives collectively present learning motivation as a dynamic interplay of internal desires, external influences, and cognitive valuation, which is crucial for understanding student engagement and achievement in educational settings.

### 2.2 Self-efficacy

Originating from social cognitive theory, the concept of self-efficacy, introduced by Bandura (1977), refers to an individual’s belief in their ability to perform tasks at a certain level. In the learning domain, self-efficacy is shaped by four primary sources identified by Bandura (1986): direct experiences, vicarious experiences, verbal persuasion, and emotional and physiological states. Seligman (1990) noted that students with developing or low self-efficacy are especially influenced by their emotional and physiological conditions. This concept encapsulates students’ confidence in their ability to complete learning tasks and profoundly impacts their engagement, choice of strategies, and academic adjustment, as highlighted by Zimmerman (1995). Robbins et al. (2004) defined self-efficacy as an individual’s evaluation of their capacity and likelihood of achieving academic success. Luszczynska et al. (2005) expanded on this, suggesting that self-efficacy is a unique belief in one’s overall ability and confidence, shaped by various environmental factors. Specifically, self-efficacy pertains to a student’s confidence in their ability to effectively complete academic tasks, which is cultivated through their experiences and achievements in educational pursuits (Fredricks et al., 2005).

### 2.3 Expectancy-value theory: bridging self-efficacy and learning motivation

In the early 1980s, psychologist Eccles and other scholars introduced the Expectancy-Value Theory, offering a framework for a deeper understanding of individual learning motivation and behavior. The core elements of the theory encompass individuals’ expectations of success and their valuation of activities. This theory is particularly relevant in educational psychology for exploring the relationship between self-efficacy and learning motivation.

Expectancy is a central concept of the theory, focusing on an individual’s confidence in successfully completing tasks, which closely aligns with the concept of self-efficacy. Self-efficacy refers to an individual’s belief in their ability to perform well in academic tasks. This confidence shapes students’ expectations of success, thereby influencing their learning motivation. High self-efficacy increases the likelihood of expecting success in academic endeavors, which is a crucial factor in stimulating motivation.

Complementing expectancy is another critical component of the theory—value. This component involves individuals’ subjective valuation of activities, which includes interest value, utility value, and achievement value. Interest value refers to the degree of personal interest in learning activities, utility value relates to the relevance of activities to personal goals, and achievement value concerns the importance of successfully completing tasks. This component suggests that when students perceive a learning activity as meaningful and useful, their willingness to engage in learning increases. Therefore, the value students place on learning activities is a key factor influencing their motivation to learn.

Under the framework of Expectancy-Value Theory, the interplay between self-efficacy and motivation becomes evident. High levels of self-efficacy boost individuals’ expectations of success, while students who value learning activities are more likely to be motivated to achieve their learning objectives. This dynamic not only fosters students’ academic achievement but also equips educators with strategies to enhance students’ self-efficacy and the perceived value of learning activities, thereby improving their motivation to learn. Therefore, understanding and applying the Expectancy-Value Theory is essential for enhancing educational practices and student academic performance.

### 2.4 Relationship between self-efficacy and motivation

There is substantial evidence indicating that self-efficacy and motivation are inextricably linked. Bandura’s (1977) research underscores self-efficacy as a key determinant of learning motivation, particularly in achievement contexts. Individuals with high self-efficacy tend to exhibit greater enthusiasm, exert more effort, and employ effective strategies in learning activities, which, in turn, reinforces their self-efficacy and sustains their motivation. For instance, Ramos Salazar and Hayward (2018) conducted a study with 160 undergraduate students at a public university, revealing that both academic and problem-solving self-efficacy predict students’ learning motivation and exam performance. Similarly, Hasanah et al. (2019) discovered that self-efficacy significantly affects motivation among 296 business administration students from vocational schools. The importance of students’ self-assurance in independent learning is crucial for success across various educational stages. Compared to their counterparts in basic education, university students demonstrate higher levels of autonomy and an increased need for self-regulation skills. Given the less supervised environment in higher education, the focus on self-efficacy becomes particularly important (Sander and Sanders, 2009). Therefore, the historical significance and contemporary relevance of self-efficacy as a concept cannot be overstated in the context of educational success.

Although most current research indicates a positive impact of high self-efficacy on motivation, some studies present an opposing view. For instance, Vancouver et al. (2002) found that under certain conditions, an increase in self-efficacy is negatively correlated with performance in subsequent trials. This negative correlation is attributed to overconfidence, which increases the likelihood of logical errors in tasks. Similarly, Moore and Healy (2008) suggest that excessively high self-efficacy can lead to overconfidence, potentially exerting a negative influence on students’ learning motivation. These findings underscore the ongoing debate within the academic community regarding the universally positive effects of high self-efficacy on motivation. Consequently, a more comprehensive and systematic investigation is needed to fully understand the complexities and nuances of this relationship.

### 2.5 Objectives and research questions

The primary aim of this systematic review is to investigate the relationship between self-efficacy and motivation among university students, focusing on studies published between 2019 and 2024. This review seeks to understand how these two variables interact within academic settings and to identify other variables frequently studied in conjunction with self-efficacy and motivation. By analyzing recent literature, this study aims to highlight current research trends, offering insights into how these constructs are conceptualized and examined in contemporary educational research.

Guided by this objective, the study seeks to address the following research questions:

1. What is the relationship between self-efficacy and motivation in the academic studies of university students published between 2019 and 2024?
2. Which additional variables are frequently studied alongside self-efficacy and motivation among university students, and how do these variables interact with self-efficacy and motivation?
3. What emerging trends are evident in the research on self-efficacy and motivation in higher education from 2019 to 2024?
4. How do these emerging trends enhance our understanding of the dynamics between self-efficacy, motivation, and related variables in the context of university education?

## 3 Methodology

### 3.1 Research design

This study employed a systematic review methodology, adhering to PRISMA (Moher et al., 2015) guidelines, to identify relevant literature on self-efficacy and learning motivation among university students. The initial literature search was conducted on December 29, 2023, using the Education Resources Information Centre (ERIC), SCOPUS, and Web of Science (WoS) databases. For SCOPUS and WoS, searches utilized specific keywords: “self-efficacy” AND “motivation” AND “university students.” In ERIC, a manual selection of relevant papers ensured the comprehensive inclusion of studies related to higher education. A final search was conducted on March 2, 2024, to incorporate the most recent publications. The search criteria were limited to open-access journals and conference papers published in English within the last five years, from 2019 to 2024, to ensure the focus remained on the most recent and pertinent studies in this field.

### 3.2 Literature selection and bias control

The search yielded a total of 5,890 documents, comprising 2,065 from Scopus, 3,464 from Web of Science, and 361 from ERIC. A search string was utilized across these three sources, including (TITLE-ABS-KEY (Motivation) AND TITLE-ABS-KEY (Self-efficacy) AND TITLE-ABS-KEY (Higher education)) AND (LIMIT-TO (PUBYEAR, 2019) OR LIMIT-TO (PUBYEAR, 2020) OR LIMIT-TO (PUBYEAR, 2021) OR LIMIT-TO (PUBYEAR, 2022) OR LIMIT-TO (PUBYEAR, 2023) OR LIMIT-TO (PUBYEAR, 2024)) AND (LIMIT-TO (LANGUAGE, “English”)) to identify relevant academic papers within the specified study parameters. The selection criteria prioritized studies examining the relationship between domain-specific self-efficacy and learning motivation among university students, with an emphasis on empirical studies published between 2019 and 2024. Duplicate records were manually removed using EndNote, and preprints were included in the analysis. However, only peer-reviewed studies were considered for in-depth evaluation, as preprints were treated as supplementary evidence, not forming the core basis of our conclusions.

The time frame of 2019 to 2024 was selected to focus on the most recent developments in the field, including the impact of the COVID-19 pandemic on student learning. This period was chosen to capture the effects of the shift toward more online and hybrid learning environments, which are particularly relevant to the relationship between self-efficacy and motivation. By restricting the search to studies published in English and peer-reviewed journals, high-quality academic research was ensured. The selection criteria also prioritized studies that provided a rigorous examination of the relationship between domain-specific self-efficacy and learning motivation among university students.

### 3.3 Quality appraisal

Because most of the included studies adopted cross-sectional survey designs, we appraised methodological quality using the Joanna Briggs Institute (JBI) Critical Appraisal Checklist for Analytical Cross-Sectional Studies (Joanna Briggs Institute, 2017). The checklist covers eight domains, including clarity of inclusion criteria, detailed description of subjects and setting, validity and reliability of exposure and outcome measures, identification and management of confounding factors, and appropriateness of statistical analysis.

Each study was rated independently by two reviewers as “yes,” “no,” “unclear,” or “not applicable” for each item. Studies with multiple “no/unclear” ratings in key domains (e.g., measurement clarity, analysis of the relationship between self-efficacy and motivation) were excluded from the final synthesis. This rigorous evaluation process resulted in 31 studies being included in the systematic review.

On the basis of the JBI checklist, studies were excluded when:

(a) The research design was not clearly specified or inappropriate for answering the review questions (*N* = 6).
(b) Key variables (self-efficacy or motivation) were not clearly defined or measured with validated instruments (*N* = 1).
(c) The analysis did not report or test the association between self-efficacy and motivation (*N* = 20).

After this appraisal, 31 studies remained for the final synthesis.

### 3.4 Initial screening and exclusion criteria

Initial screening excluded documents based on type, removing book chapters (*N* = 49), books (*N* = 21), data papers (*N* = 1), editorial material (*N* = 4), and retracted publications (*N* = 1). Further screening for relevance, language, and abstract content led to the exclusion of 5,814 records. The specific reasons for exclusion were: not relevant to higher education (*N* = 1,543), not written in English (*N* = 227), lack of abstract (*N* = 4), and not related to the proposed research questions (*N* = 3,982). This process narrowed the selection to 58 full-text articles for detailed eligibility assessment.

**Figure 1:**
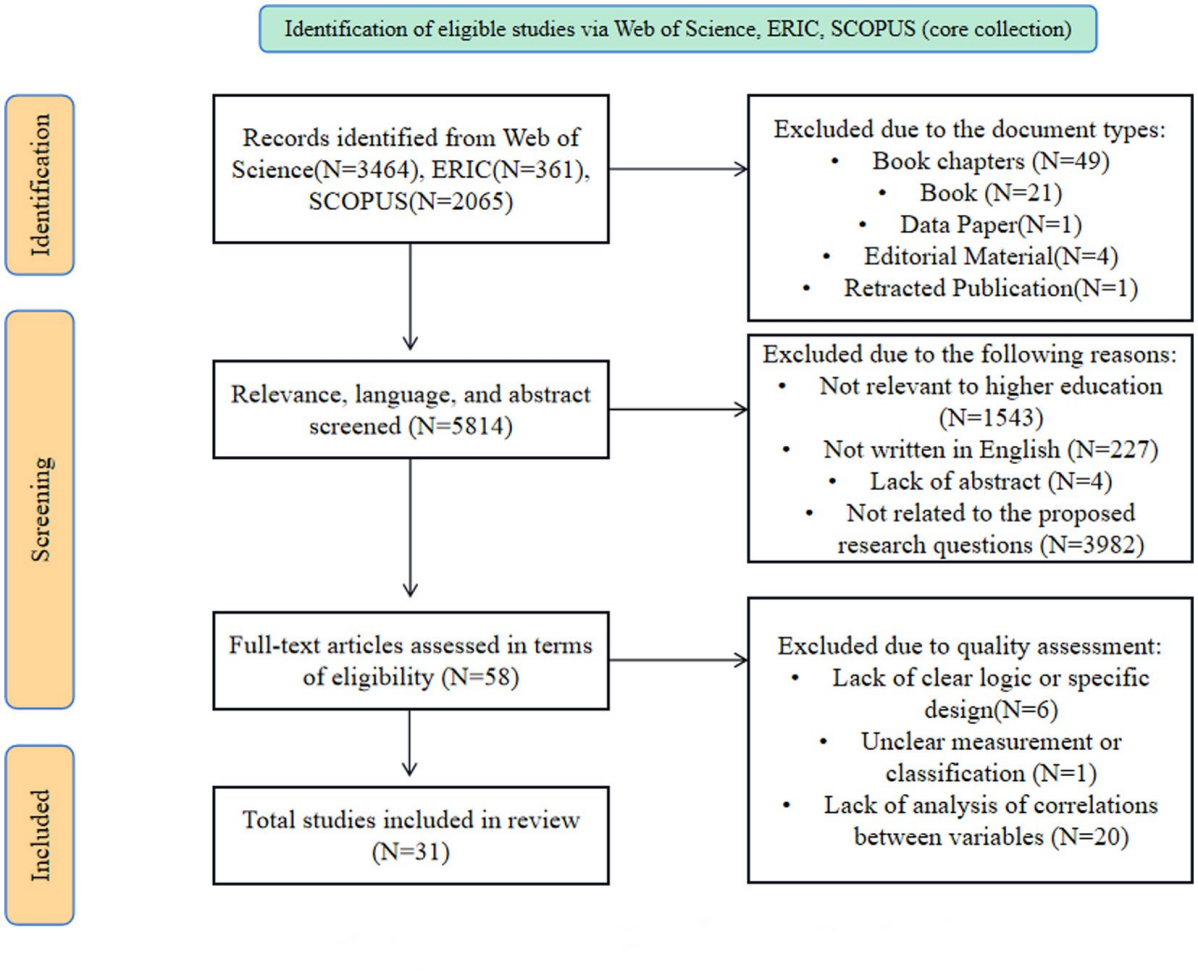
Flow chart process of the current review.

The assessment of these full-text articles resulted in additional exclusions based on quality assessment criteria: lack of clear logic or specific design (*N* = 6), unclear measurement or classification (*N* = 1), and lack of analysis of correlations between variables (*N* = 20). This rigorous evaluation process resulted in 31 studies being included in the systematic review. The flowchart of the current review is shown below in Figure 1.

The chosen time frame of 2019 to March 2024 was selected to include papers published in English, peer-reviewed journal articles, and unpublished papers. This period was chosen to capture the most recent and relevant research findings in the field. By including the latest developments and emerging trends, this review aims to reflect current knowledge and practices. Additionally, focusing on recent publications allows for an examination of the impact of global events, such as the COVID-19 pandemic, providing insights into how these events have influenced research directions and priorities. The sampling population was restricted exclusively to university students, with no exclusions based on age, gender, or other demographic factors. When studies included multiple predictor and outcome variables, only those articles investigating the relationship between domain-specific self-efficacy and learning motivation were included.

## 4 Results

### 4.1 Research trends

**Figure 2:**
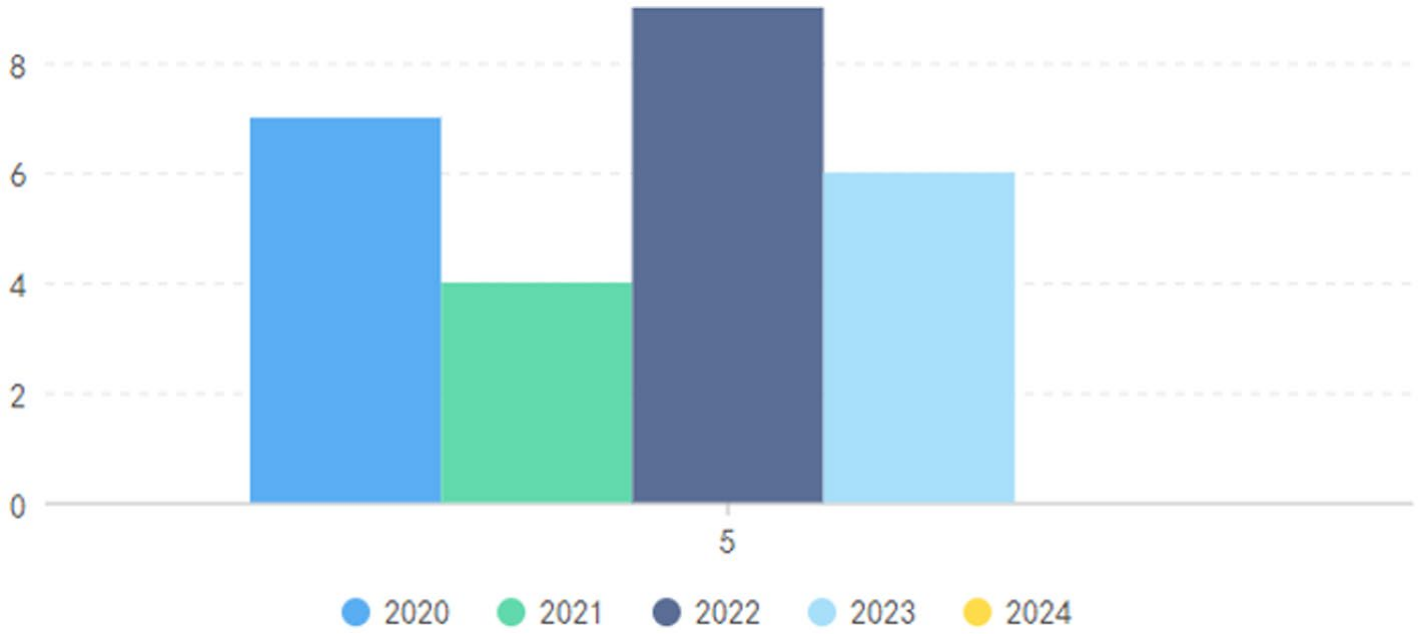
The number of documents published annually.

To examine significant contributions and identify research trends within this specialized domain, a bibliometric analysis was conducted, encompassing various dimensions. The investigation included titles, authors, participants, publication years, variables studied, sources of publication, types of documents, geographic locations, author affiliations, research methodologies, and key findings. Figure 2 illustrates the annual distribution of published documents, highlighting fluctuating scholarly interest over the years. Specifically, the analysis shows the publication of 5 documents in 2019, 7 in 2020, 4 in 2021, 9 in 2022, 6 in 2023, and none in 2024 thus far. This temporal distribution indicates the evolving nature of research in this field and the scholarly community’s responsive engagement with the themes of self-efficacy and motivation among university students.

**Figure 3:**
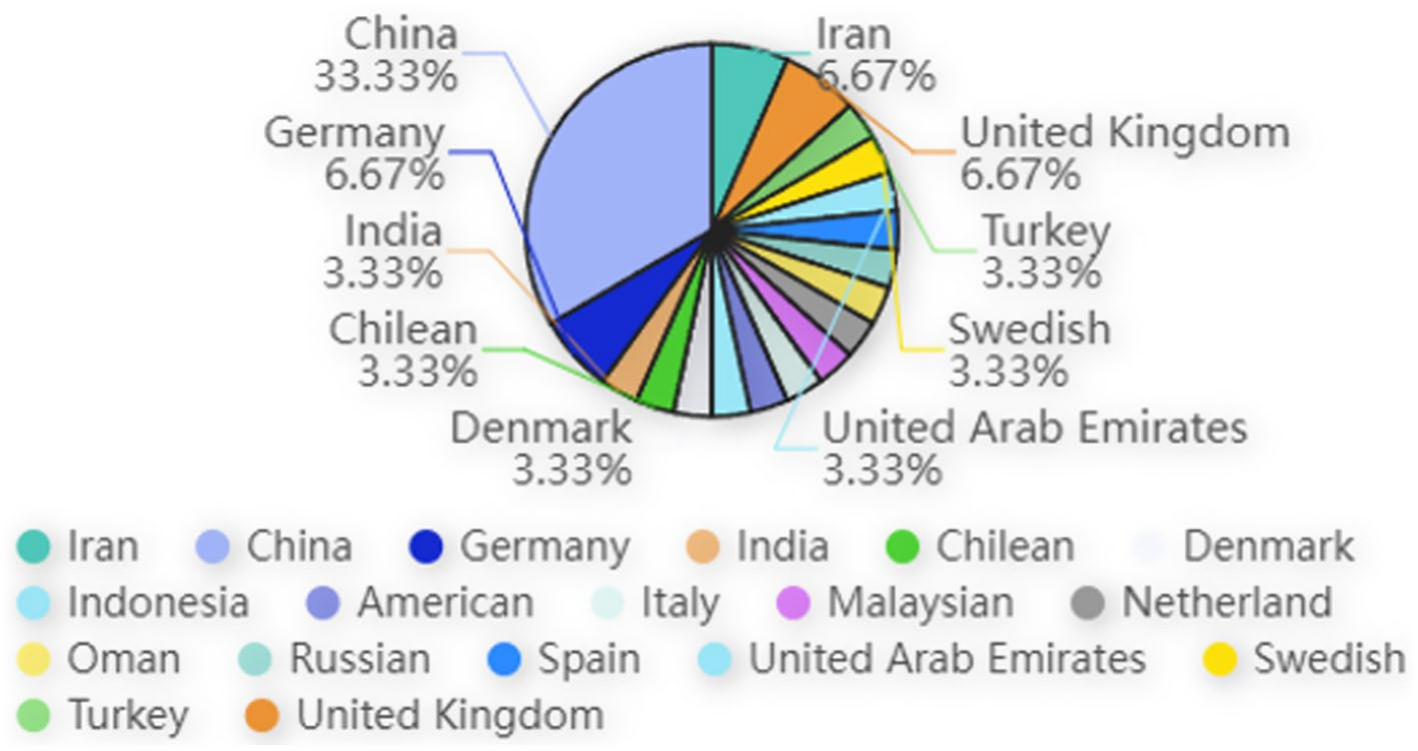
Contribution of different countries.

This review encompasses a total of 31 documents, including 29 journal articles, 1 conference paper, and 1 preprint. The distribution of publishers reveals a diverse array of academic sources, with the conference paper disseminated by one publisher and 28 distinct publishers contributing to the journal articles. Geographically, the studies offer a global perspective, with a significant portion—approximately 33.33%—originating from China, highlighting the substantial contribution of Chinese scholarship to this field. Additionally, the research includes studies conducted in various countries, with two studies each from Germany and Iran, and one study each from the United Kingdom, Australia, India, Chile, Denmark, Indonesia, Italy, Malaysia, the Netherlands, Oman, Russia, Spain, the United Arab Emirates, Sweden, the United States, and Turkey. This worldwide distribution underscores the universal relevance and interdisciplinary nature of the research theme, with the majority of studies conducted in Asia. Figure 3 provides a graphical representation of these findings, illustrating the geographical spread and frequency of contributions within this academic inquiry.

In terms of author affiliations, the review has identified a diverse array of academic institutions from various countries, reflecting the international scope of the research. The affiliations encompass 26 unique universities and 5 research institutions. Notably, several countries, such as Malaysia and Iran, are represented by multiple institutions, highlighting strong regional research interests. For instance, in Malaysia, institutions such as UOW Malaysia KDU University College, Universiti Utara Malaysia, and the University of Malaya are represented, with the University of Malaya mentioned three times, indicating its significant contribution to the field. The geographic distribution of these affiliations is broad, spanning Asia with universities like the National Taiwan University of Science and Technology and Peking University, the Middle East with institutions like Sultan Qaboos University in Oman, Europe with institutions such as Saarland University in Germany, and other regions including Edith Cowan University in Australia. This global distribution underscores the universal relevance and interdisciplinary nature of the research theme.

Several universities have multiple departments or faculties listed, reflecting a multidisciplinary approach to the research topics. For example, King Saud University in Saudi Arabia has its College of Nursing mentioned three times, suggesting a concentrated research effort in this area.

**Figure 4:**
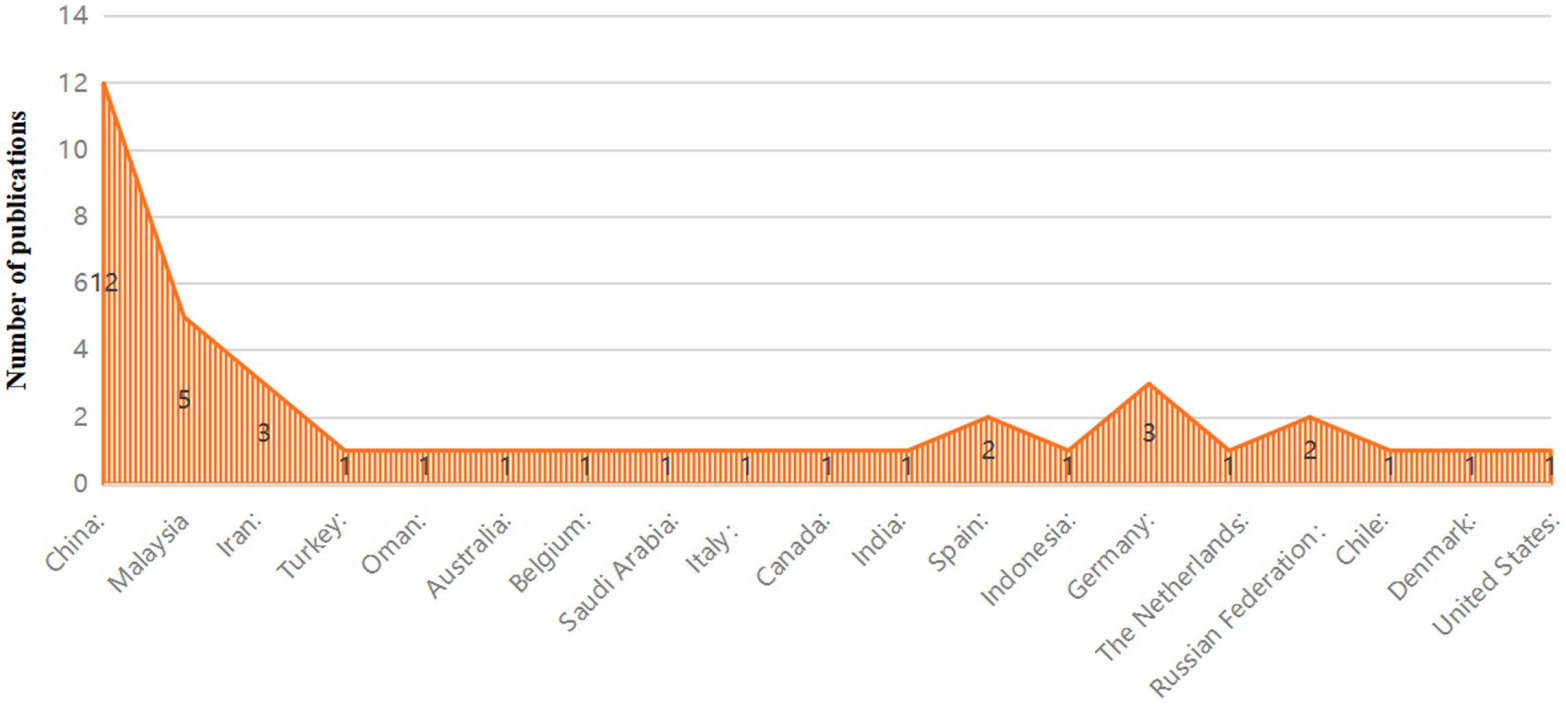
Contribution of author affiliations.

Figure 4 presents the contributions of author affiliations, providing a visual overview of the global academic landscape involved in this area of research.

### 4.2 Relationship between domain-specific self-efficacy and motivation

The literature consistently acknowledges a positive and significant relationship between domain-specific self-efficacy and motivation. A summary of the literature can be seen in Table 1. Specifically, of the studies reviewed, a majority (15 out of 24) unequivocally support the notion that higher levels of self-efficacy correlate with increased motivation. Additionally, the synthesis reveals a nuanced understanding of how self-efficacy operates in various capacities beyond direct correlation. Six studies (2, 5, 8, 9, 16, 25) elucidate the mediating role of self-efficacy, showing how it bridges the gap between educational practices (such as classroom climate and learning strategies) and motivation. This underscores the importance of nurturing students’ belief in their abilities as a crucial pathway to enhanced academic engagement.

**Table 1:** A summary of the literature.

No.	Author	Year	Size	Method	Relationship between self-efficacy and academic motivation	Mediating/Moderating variable
1	Abdolrezapour et al.	2023	120	Quantitative	Significant positive correlation (*r* = 0.36; *p* < 0.01)	None listed
2	Qiumei Wang et al.	2022	446	Quantitative	Positive relationship (*β* = 0.460; *p* = 0.000)	Mediating effect (*β* = 0.195; *p* = 0.000)
3	Aline Salzmann et al.	2022	92	Quantitative	Significant positive correlation (rho = 0.553; *p* < 0.001)	None listed
4	Yong Shee Mun et al.	2022	535	Mixed-methods	No causal link found	None listed
5	Lekissa Alemayehu, et al.	2021	354	Quantitative	Stronger correlation (*ß* = 0.79; *p* < 0.001)	Partial mediation
6	Amoozegar et al.	2022	303	Quantitative	Supported all direct relationships	None listed
7	Jonathan Álvarez Ariza	2022	44	Mixed-methods	Positive correlation with motivation	None listed
8	Qiumei Wang et al.	2020	119	Quantitative	Significant positive relationship	Mediating effect
9	Mahmut Polatcan	2023	545	Quantitative	Partial mediating role in the relationship	Partial mediation
10	ElAdl Adel et al.	2020	196	Quantitative	Generally positive significant correlation	None listed
11	McClusky et al.	2023	43	Mixed-methods	Self-efficacy influenced motivation levels	None listed
12	Duchatelet et al.	2019	230	Quantitative	Significant positive correlation (*ß* = 0.560; *p* < 0.001)	Moderating effect
13	Bahari Ghareeb et al.	2022	110	Quantitative	Strong, positive significant correlation (*r* = 0.663; *p* < 0.001)	None listed
14	Pedditzi et al.	2019	100	Quantitative	Predictive of achievement motivation	None listed
15	Wu et al.	2020	1930	Quantitative	Both intrinsic and extrinsic motivation predicted self-efficacy	None listed
16	Pan	2020	332	Quantitative	Motivation mediated the relationship	Mediation effect
17	Aboobaker et al.	2023	330	Quantitative	Indirect effect on entrepreneurship intention, moderated by technological self-efficacy	Moderating effect
18	Lin et al.	2021	39	Mixed-method	Significantly enhances intrinsic motivation	None listed
19	Alvarez-Huerta et al.	2019	710	Quantitative	Related to student motivation and learning	None listed
20	Haerazi et al.	2020	120	Quantitative	Significant positive correlation	None listed
21	Johannes et al.	2022	75	Quantitative	Motivation directly influenced knowledge gain and self-efficacy changes	None listed
22	Abdolrezapour et al.	2023	120	Quantitative	Significant positive relationship	None listed
23	Yin et al.	2021	256	Quantitative	Directly and positively related	None listed
24	Hsia et al.	2020	65	Quantitative	Significant positive correlation	None listed
25	Deng et al.	2022	203	Quantitative	Fully mediates the association	Full mediation effect
26	Ilishkina et al.	2022	716	Quantitative	Significant relationships in some instances	None listed
27	Spittle et al.	2023	291	Quantitative	Significantly related	None listed
28	Díaz Mújica et al.	2019	2,741	Quantitative	Positively related variables	None listed
29	Makransky et al.	2019	199	Quantitative	Positive relation to changes in self-efficacy	None listed
30	Wang et al.	2020	475	Quantitative	Positive correlation	None listed
31	Peng et al.	2021	262	Quantitative	Positive influence on motivation	None listed

In addition to mediation, the review identifies two studies (12, 17) that position self-efficacy as a moderating factor. These studies suggest that the impact of motivational strategies or external interventions on student motivation can vary based on the level of self-efficacy. This finding implies that the effectiveness of certain educational interventions may depend on students’ self-belief, highlighting the need for tailored motivational approaches that account for individual differences in self-efficacy.

Conversely, only one study (4) reports the absence of a significant relationship, presenting an anomaly that may be attributed to specific contextual factors. This outlier underscores the necessity for further investigation to understand the conditions under which self-efficacy might not relate to motivation.

**Figure 5:**
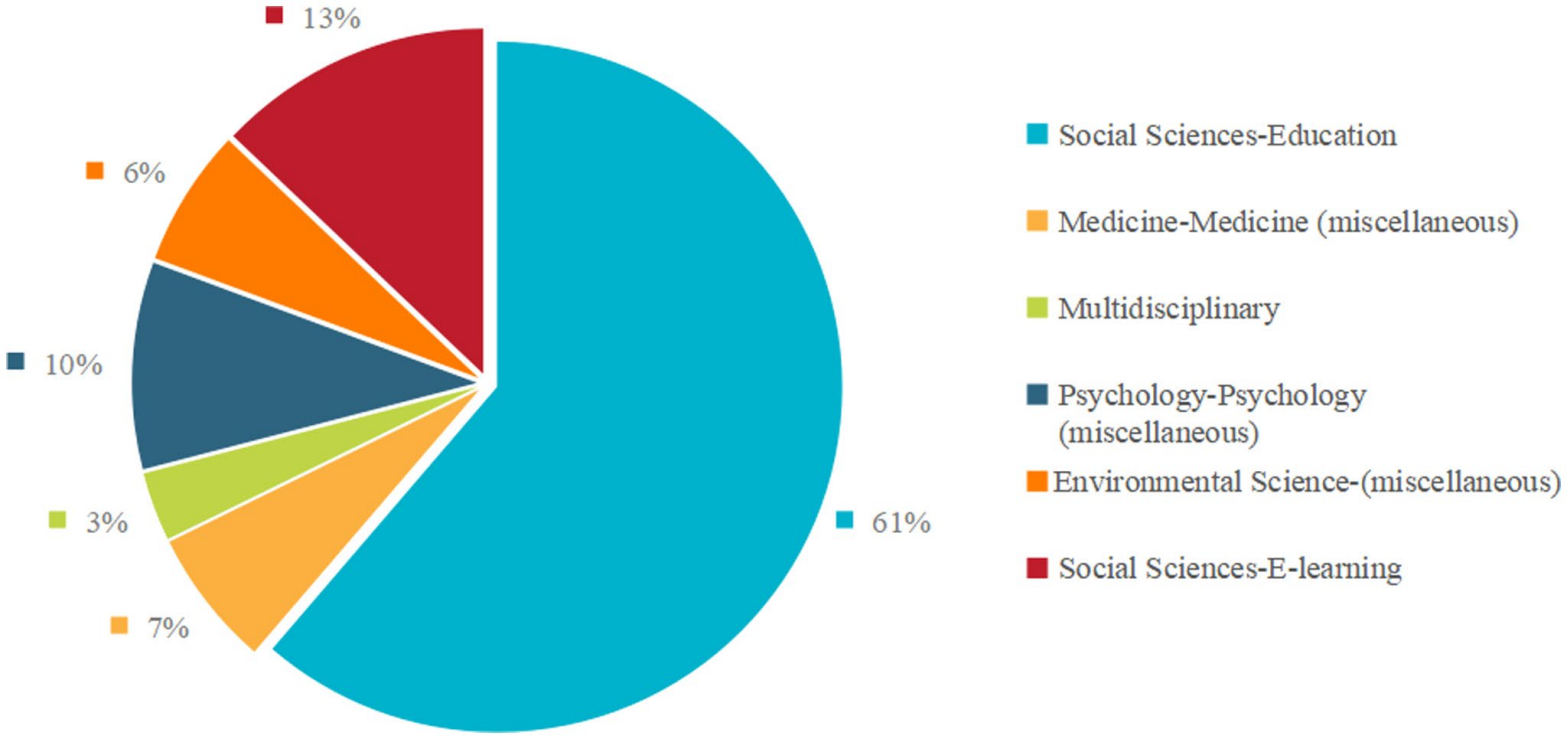
Fields of research.

An examination of recent literature, as illustrated in Figure 5 and detailed in Table 1, reveals a strong focus on exploring the relationship within the Social Sciences-Education sector, which accounts for 61% of the studies. This is followed by research in the Social Sciences-E-learning context at 13%, highlighting the growing importance of digital platforms in learning environments. The Psychology-Psychology (miscellaneous) domain contributes 10% of the studies, while Medicine-Medicine (miscellaneous) and Environmental Science (miscellaneous) each account for 6 and 7%, respectively. Although a multidisciplinary approach is present, it is less prominent, comprising 3% of the studies.

**Figure 6:**
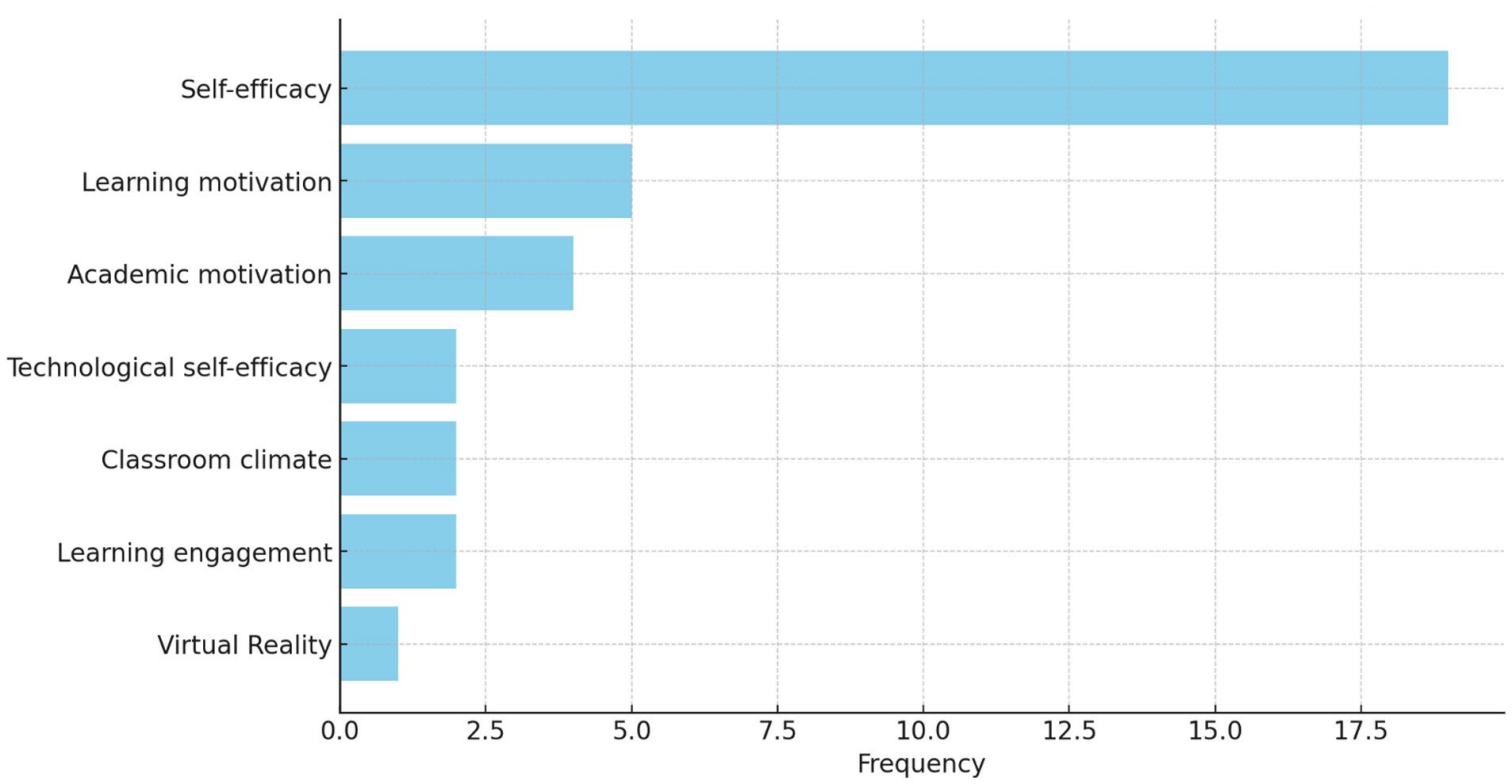
Frequency of variables in academic research (Last 5 Years).

From Figure 6, we can conclude that over the past 5 years, 31 papers have consistently addressed the concepts of self-efficacy and motivation among university students, highlighting their significance in academic learning. Self-efficacy emerged as the most frequently mentioned variable, cited 19 times across various studies (Díaz Mujica et al., 2019; Wang and Zhan, 2020; Peng and Hwang, 2021; Yin and Yuan, 2021; Álvarez Ariza, 2022; Amoozegar et al., 2022; Deng et al., 2022; Ilishkina et al., 2022; Abdolrezapour et al., 2023; Alemayehu and Chen, 2023; Spittle et al., 2023), underscoring its pivotal role in student learning processes. Learning motivation was discussed in five separate papers (Pan, 2020; Bahari et al., 2022; Shee and Lip, 2022; Aboobaker et al., 2023; Alemayehu and Chen, 2023), reflecting researchers’ interest in understanding the intrinsic forces that drive students through their academic endeavors. These studies encompass a range of learning environments, from online to face-to-face settings, and various disciplines, from medical education to engineering, suggesting the universal importance of learning motivation across different fields of education.

On the other hand, academic motivation, mentioned four times (Wang et al., 2020; Abdolrezapour et al., 2023; Wang et al., 2023), is closely linked to students’ academic achievements, enduring learning pursuits, and overall satisfaction with their academic experiences. The research identified various methods and influencing factors for enhancing academic motivation, such as teacher leadership styles, classroom climate, and their interaction with students’ self-efficacy. Furthermore, the studies discussed enhancing students’ motivation through educational practices, curriculum design, and teacher training. For instance, Shee and Lip (2022) explored how the learning environment and teacher-student interactions impacted online learning motivation during the COVID-19 pandemic. Alemayehu and Chen (2023) examined how motivation could influence learning engagement through self-monitoring and self-efficacy. These findings emphasize the roles of external factors and internal psychological processes in the development of motivation.

In addition, technological self-efficacy was mentioned twice, underscoring the role of technology in learning motivation and the increasing focus on this area within educational technology research (Pan, 2020; Aboobaker et al., 2023). Classroom climate was also highlighted twice (Wang et al., 2020, 2023), as was learning engagement (Wu et al., 2020; Alemayehu and Chen, 2023), indicating the significant impact of educational environments and practices on students’ self-efficacy and motivation. Although virtual reality was mentioned less frequently, its exploration in one study (Lin and Wang, 2021) reflects a growing interest in the application of emerging technologies in education.

## 5 Discussion

RQ1: What is the relationship between domain-specific self-efficacy and motivation in the academic studies of university students published between 2019 and 2024?

This systematic review has highlighted the intricate relationship between self-efficacy and motivation within the context of higher education, underscoring their pivotal role in the academic journey of university students. The findings reveal a predominantly positive correlation between domain-specific self-efficacy and motivation, reinforcing the idea that students with a strong belief in their capabilities are more likely to exhibit higher levels of motivation in their academic pursuits. This relationship aligns with Bandura’s social cognitive theory, which identifies self-efficacy as a central determinant of motivation and behavior (Bandura, 1977). Additionally, the review highlights the mediating and moderating roles of self-efficacy, suggesting its dynamic functionality in influencing academic motivation. Self-efficacy not only directly impacts motivation but also acts as a crucial link between various educational practices and students’ motivational levels. This pattern is broadly consistent with Dogan (2015), who showed that both academic self-efficacy and academic motivation were significant predictors of students’ academic performance in a school context. In Dogan’s study, self-efficacy and motivation operated alongside cognitive engagement as independent predictors, rather than as mediating variables linking instructional factors to performance. These insights are invaluable for educators and policymakers, emphasizing the necessity of creating environments and implementing interventions that bolster students’ self-efficacy to enhance their motivation and, consequently, their academic success.

RQ2: Which additional variables are frequently studied alongside self-efficacy and motivation among university students, and how do these variables interact with self-efficacy and motivation?

This review identifies several additional variables frequently studied alongside self-efficacy and motivation, including academic motivation, classroom climate, teacher leadership, and technological self-efficacy. These variables were found to influence the relationship between self-efficacy and motivation in various ways. For instance, academic motivation is closely linked to long-term academic achievements and student satisfaction (Wang et al., 2020; Abdolrezapour et al., 2023). Teacher leadership styles and classroom climate also significantly impact self-efficacy and motivation by shaping students’ expectations of success and the perceived value of learning tasks. These external variables enhance students’ intrinsic motivation, supporting Self-Determination Theory (Ryan and Deci, 2000), which highlights the role of supportive environments in fostering intrinsic motivation.

Moreover, technological self-efficacy has become increasingly important in online learning contexts. As technology plays a central role in modern education, students’ belief in their ability to use digital tools effectively has a direct impact on their motivation (Pan, 2020; Aboobaker et al., 2023). The growing integration of technology in education highlights the relevance of this variable in the current research landscape.

However, the interaction between these variables and self-efficacy is complex and dynamic. For example, teacher-student interactions, as discussed by Shee and Lip (2022), are particularly important in online learning environments, where direct social interactions are limited. In these settings, the classroom climate and teacher support become crucial in maintaining students’ motivation and engagement, especially when combined with strong self-efficacy. According to Expectancy-Value Theory (EVT), students’ motivation is significantly influenced by their expectation of success and the value they place on the task (Eccles et al., 1983). In online learning environments, the perceived value of academic tasks can be enhanced by positive teacher-student interactions and a supportive classroom climate, while the expectation of success is shaped by students’ self-efficacy and teacher feedback. These findings highlight the importance of both external and internal factors in motivating students to engage with academic tasks.

RQ3: What emerging trends are evident in the research on self-efficacy and motivation in higher education from 2019 to 2024?

An emerging trend in the literature is the growing focus on the role of online and hybrid learning environments. With the shift toward digital learning in response to the COVID-19 pandemic, many studies have examined the impact of technological self-efficacy on student motivation in online settings. This is particularly relevant as students with higher technological self-efficacy tend to engage more actively in online learning environments. These findings underscore the importance of considering the evolving nature of education, where digital tools and platforms play an increasingly significant role in shaping students’ motivation and self-efficacy.

RQ4: How do these emerging trends enhance our understanding of the dynamics between self-efficacy, motivation, and related variables in the context of university education?

The trends identified in this review indicate that the relationship between self-efficacy and motivation is not static but is influenced by various contextual factors, including the learning environment and teaching practices. The increasing prevalence of online and hybrid learning models calls for a more nuanced understanding of how technological self-efficacy interacts with students’ motivation. Furthermore, the review suggests that enhancing students’ self-efficacy, both in academic tasks and in using digital technologies, can foster greater motivation, particularly in a rapidly changing educational landscape. These insights are valuable for educators and policymakers aiming to design interventions that improve both the self-efficacy and motivation of students, particularly in digital learning environments.

However, the review also identifies a notable exception in the literature, where a study reported no significant relationship between self-efficacy and motivation (Shee and Lip, 2022). This outlier highlights the complexity of human behavior and the impact of contextual factors on psychological constructs. It suggests that the relationship between self-efficacy and motivation might not be universally consistent across different settings or disciplines. This invites further investigation into the specific conditions under which self-efficacy might not predict motivation, potentially revealing deeper insights into the nuanced nature of these constructs and their interactions.

## 6 Conclusion

The multidisciplinary nature of the research, spanning fields such as social sciences, psychology, and medicine, underscores the broad applicability and significance of self-efficacy and motivation across various domains of human activity. This highlights the potential for interdisciplinary research to provide comprehensive insights into the factors that influence academic motivation and the strategies that can enhance it. The relationship between self-efficacy and motivation is complex and multifaceted, influenced by numerous factors including educational practices, individual differences, and contextual variables. This complexity underscores the need for continued research, particularly studies that seek to unravel the contextual and cultural dimensions influencing the efficacy-motivation nexus. This review extends Bandura's (1977) social cognitive theory by incorporating contemporary challenges such as the shift to online learning environments, particularly in the wake of the COVID-19 pandemic, which have altered the ways in which self-efficacy and motivation interact in educational contexts.

The geographical distribution of the studies in this review indicates a global interest in the dynamics of self-efficacy and motivation, with significant contributions from various regions, particularly Asia. This diversity enriches the understanding of these constructs, enabling a cross-cultural examination of their applicability and relevance across different educational systems and contexts. By synthesizing findings from various global contexts, this review refines Expectancy-Value Theory (Eccles et al., 1983) by highlighting the evolving nature of task value and success expectations in modern educational settings, especially within online and hybrid learning environments. The study also introduces cultural factors into the framework, demonstrating that motivation in certain regions may be shaped by different contextual and educational influences. It emphasizes the universal importance of self-efficacy and motivation in educational discourse, transcending cultural and regional boundaries. Overall, the global scope of the reviewed studies highlights a widespread interest in the interplay between self-efficacy and motivation across educational contexts. The notable contributions from Asia underscore the universal relevance of these constructs, facilitating a cross-cultural understanding of their impact within diverse educational systems.

Additionally, there are clear implications for educational practitioners to develop targeted interventions that enhance students’ self-efficacy while considering the broader motivational landscape in which students operate. This review also extends theories of self-efficacy and motivation by suggesting that interventions should not only focus on individual traits but also account for external influences, such as classroom climate and teacher-student interactions, which have been shown to impact both self-efficacy and motivation in unique ways across different learning environments. By addressing these areas, educators can more effectively support students in their academic endeavors, fostering environments conducive to learning and personal development. This systematic review highlights the critical interplay between self-efficacy and motivation within the academic setting, offering valuable insights for educators, researchers, and policymakers. By continuing to explore this relationship and its underlying mechanisms, the academic community can contribute to the development of more effective educational strategies that support student success and well-being.

### 6.1 Limitations of the study

This systematic review has several limitations that should be acknowledged:

1. Geographical and Cultural Scope: The majority of included studies originate from Asia, which may bias the findings toward these specific educational systems and cultural contexts. Consequently, this may limit the generalizability of the conclusions to other regions and cultural settings.
2. Language and Methodology Bias: The review is limited to articles published in English and primarily focuses on quantitative studies. This exclusion of relevant research published in other languages or qualitative studies may result in a lack of deeper insights into the constructs of self-efficacy and motivation.
3. Influence of Other Moderating and Mediating Variables: The review does not extensively explore additional variables that could influence the relationship between self-efficacy and motivation, such as socio-economic status, institutional factors, or other moderating and mediating variables. This may oversimplify the complex dynamics at play and limit the comprehensiveness of the findings.

### 6.2 Future recommendations

In light of the comprehensive insights garnered and the identified limitations within this systematic review, future research should adopt a multifaceted approach to deepen the understanding of self-efficacy and motivation among university students. Given the gap highlighted by a study that found no significant relationship between self-efficacy and motivation, it is crucial to explore the contextual and disciplinary boundaries within which this phenomenon may vary. Expanding research to include diverse cultural and educational contexts will be essential for broadening the applicability of findings. Moreover, integrating qualitative methodologies alongside quantitative analyses can provide a richer, more nuanced understanding of how students perceive and experience self-efficacy and motivation. Investigating these constructs in the context of contemporary educational challenges, such as online learning environments and the impact of global crises, will offer valuable insights. This holistic approach will not only address current gaps in the literature but also guide the development of targeted educational strategies and interventions, ultimately fostering supportive learning environments for students worldwide.

## Data Availability

The original contributions presented in the study are included in the article/supplementary material, further inquiries can be directed to the corresponding author.
